# Salivary Fistula

**Published:** 1852-04

**Authors:** 


### ARTICLE IX.

### *Salivary Fistula.*

MR. LYNCH relates the following case in the Medical Times. Anne Jane Bushell, aged five years, was brought to me for an abscess in her cheek. She had a fall on a fender about a month previously. Shortly afterwards a tumor formed, which burst, and continued to discharge a thin purulent liquid. From the nature of the discharge, and the situation of the wound, I suspected an injury of Stenon's duct, and was confirmed in this opinion by laying her on the opposite side, and noticing the cavity of the wound, after being sponged out, to fill with salivary fluid. Having consulted with Mr. Chalmers, of Everton, we adopted the following mode of procedure: I introduced a small hydrocele trocar and canula through the wound, from without inwards, and, having got it into the mouth, withdrew the trocar, leaving the canula, having previously formed a piece of gutta percha to fit the canula, about two inches long, with a head to it, somewhat resembling a twopenny tack with the point cut off. Through the shoulder of the apparatus, I passed some ligature silk, and, by means of an eye probe, I passed the silk through the canula, and then withdrew the latter, securing the threads with some strips of adhesive plaster to the cheek beyond the abscess, thus forming an efficient seton; the head on the interior of the cheek enabling me to fix it firmly. After the seton had remained in the cheek about five weeks, I was obliged to withdraw it, owing to its causing great swelling and inflammation of the side of the face, which speedily disappeared, and the saliva for some days passed entirely into the mouth; but in about a week after the withdrawal of the seton, it again collected, and burst externally. I now commenced to pass a small probe from the mouth in the track of the seton daily, and was obliged to continue to do so for nearly three months, before I could confidently expect a perfect cure. If a day or two was omitted, symptoms of a fresh filling of the old cyst were sure to present themselves. However, after persevering for the aforementioned length of time, I was gratified in effectually curing the fistula, as some two months have now elapsed since the last passing of a probe, and no return of the complaint has appeared. I may add, that the treatment of this seemingly trifling affection required almost daily attention for nearly five months.

